# Enhancing conventional chemotherapy drug cisplatin-induced anti-tumor effects on human gastric cancer cells both *in vitro* and *in vivo* by Thymoquinone targeting PTEN gene

**DOI:** 10.18632/oncotarget.20721

**Published:** 2017-09-08

**Authors:** Jingjing Ma, Xue Hu, Jiao Li, Dandan Wu, Qingzhi Lan, Qian Wang, Shan Tian, Weiguo Dong

**Affiliations:** ^1^ Department of Gastroenterology, Renmin Hospital of Wuhan University, Wuhan, Hubei Province, China; ^2^ Key Laboratory of Hubei Province for Digestive System Disease, Wuhan, Hubei Province, China; ^3^ Central Laboratory, Renmin Hospital of Wuhan University, Wuhan, Hubei Province, China

**Keywords:** thymoquinone, cisplatin, gastric cancer, PTEN, drug sensitivity

## Abstract

Combination chemotherapy regimen with several anti-tumor drugs is a strategy to improve outcome. Thymoquinone (TQ) has been reported to exert biological activity on various types of human cancers without obvious toxicity. However, only few studies showed the anti-tumor effects of TQ combination with cisplatin on gastric cancer (GC). Here, we showed pretreatment with 5μM TQ significantly increased the apoptotic effects induced by cisplatin on GC cell lines. Combined treatment of cisplatin with TQ represented a significantly superior tumor suppression effect than either agent alone in a xenograft tumor mouse model. Interestingly, TQ pretreatment following cisplatin caused a significant increase in the levels of PTEN, an obvious decrease in p-AKT, CyclinD1, P-glycoprotein (P-gp), meanwhile, TQ and cisplatin also led to an increase in Bax, Cyt C, AIF, cleaved caspase 9, and cleaved caspase 3, and a decrease in Bcl-2, procaspase-9, procaspase-3. Moreover, results *in vitro*, showed that a combination of TQ and cisplatin represents a more effective anti-tumor agent than either agent alone in a xenograft tumor mouse model. In conclusion, TQ significantly augments cisplatin-induced anti-tumor effects on gastric cancer both *in vitro* and *in vivo*, through inhibiting PI3K/AKT signaling pathway, activating the mitochondrial pathway, and down-regulating P-glycoprotein by up-regulating PTEN gene. TQ might be as a promising candidate as a cancer chemopreventive or chemotherapeutic agent for antineoplastic combination therapy and merits further clinical investigation.

## INTRODUCTION

Gastric carcinoma (GC) remains the one of most malignant tumors, with 1.3 million (1.2-1.4million) incident cases and 819,000 deaths 95%UI, 795 000-844 000) worldwide in 2015 [1]. GC patients were usually diagnosed at advanced stages, which compromised the effects of surgery and radiation greatly. Conventional chemotherapeutic drugs have yielded clinical benefits for GC patients for decades, whereas the clinical outcomes were quite disappointing due to drug resistance and cell toxicity [2, 3]. Here, we conduct this report depicting the application of novel chemotherapeutic agents and combinations of regimens in order to provide an improved therapy for advanced GC.

Thymoquinone (TQ, 2-isopropyl-5-methyl-1,4-quinone), is a bioactive component of Nigella sativa, which has been reported that it induces antioxidant, anti-inflammatory and anti-cancer effects [4–6]. Previous studies revealed the increasing TQ concentrations resulted in a significant inhibition of viability in various types of cancers [7–9]. TQ, exhibits anti-tumor activity via various mechanisms, specifically by interfering with DNA structure, effecting numerous carcinogenic signaling pathways and inducing immunomodulation [10–13]. An interesting research focusing on resistant breast cancer cells, revealed the effect of TQ on cell signaling and survival pathways by up-regulating expression of PTEN and then inhibiting the PI3K/AKT signaling pathway [14]. Moreover, when used in combination with 5-fluorouracil, TQ augments its apoptotic activity in colon cancer *in vitro* and *in vivo* [15]. TQ seems to be as a promising candidate as a cancer chemopreventive or chemotherapeutic agent.

To date, our research team has demonstrated that TQ inhibits the proliferation of gastric cancer cells *in vitro* and *in vivo* [16]. Here, we further explore the antitumor effects of TQ combined with cisplatin on gastric cancer cells as well as the underlying biological mechanisms.

## RESULTS

### TQ sensitizes GC cells to cisplatin-induced growth inhibition, which might be reversed by down-regulation of PTEN

**Figure 1:**
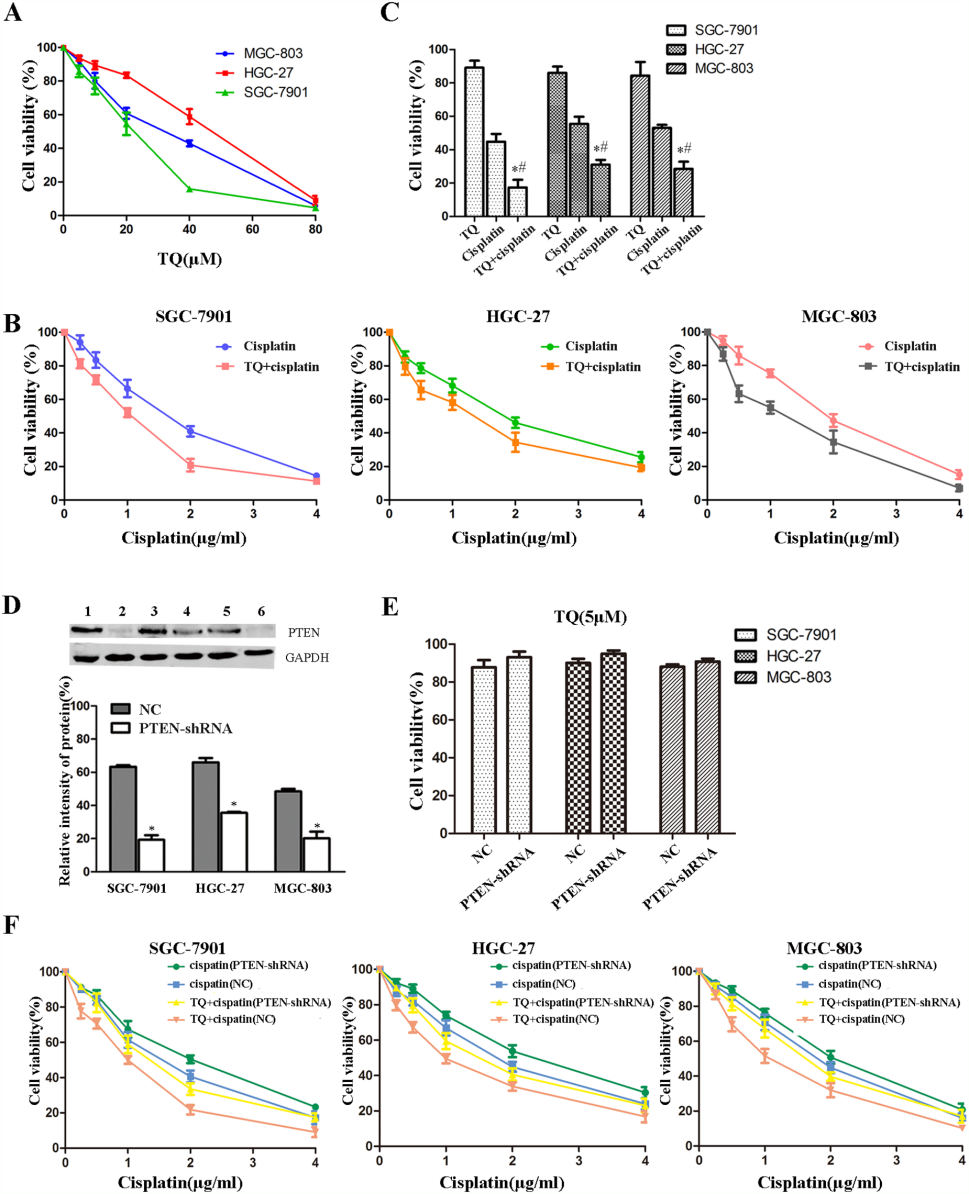
Evaluation of GC cells growth inhibition induced by TQ and/or cisplatin by CCK-8 kit **(A)** Cells were treated with TQ at different concentrations (0, 5, 10, 20, 40, 80μM) for 24h. **(B)** Cells were treated with cisplatin (0, 0.25, 0.5, 1, 2, 4 μg/ml) and a combination of 5μM TQ pretreatment + cisplatin (0, 0.25, 0.5, 1, 2, 4μg/ml) for 24h, respectively. **(C)** Cells were treated with 5μM TQ, 2μg/ml cisplatin and a combination of 5μM TQ pretreatment + 2μg/ml cisplatin for 24h. ^*^P<0.05 versus treated single 2μg/ml cisplatin group cells, ^#^*P*<0.05 versus cells treated with single 5μM TQ. **(D)** Western blotting was conducted to identify the effects of stable transfection of PTEN in GC cells. Line 1 SGC-7901/NC; Line 2 SGC-7901/PTEN-shRNA; Line 3 MGC-803/NC; Line 4 MGC-803/PTEN-shRNA; Line 5 HGC-27/NC; Line 6 HGC-27/PTEN-shRNA. ^*^P<0.05, compared with paired negative control (NC) groups. **(E)** Transfected GC cells were treated with TQ (5μM) for 24h. **(F)** Transfected GC cells were treated with cisplatin (0, 0.25, 0.5, 1, 2, 4μg/ml), and a combination of 5μM TQ pretreatment + cisplatin (0, 0.25, 0.5, 1, 2, 4μg/ml), respectively. All the above data are mean±SD from the average of three experiments.

GC cells including SGC-7901, HGC-27, MGC-803, were incubated with TQ (0, 5, 10, 20, 40, 80μM) and cisplatin (0, 0.25, 0.5, 1, 2, 4, 8μg/ml) at different concentrations for 24h respectively. TQ and cisplatin exhibited inhibition of cell growth by CCK-8 assay, respectively, in a concentration-dependent manner (Figure 1A, 1B). In addition, it was observed that of the three gastric cancer cell lines, SGC-7901 was the most sensitive to TQ. Thus, SGC-7901 was chosen for the following experiment (Figure 3–6). TQ, at a concentration up to 5μM, was of no obvious cytotoxicity with approximately 90% of cells viability in all GC cell lines tested. However, shown in the Figure 1B, GC cells pretreated with TQ (5μM), were more sensitively to cisplatin (0, 0.25, 0.5, 1, 2, 4μg/ml), the IC50 values of cisplatin in combination with TQ (5μM) in GC cells were much lower than of cisplatin alone (Table 1). Furthermore, as clearly shown in Figure 1C, TQ (5μM) pretreatment following cisplatin (2μg/ml), resulted in a significant decrease in GC cells viability compared with cisplatin alone (^*^P<0.05, respectively).

**Figure 2:**
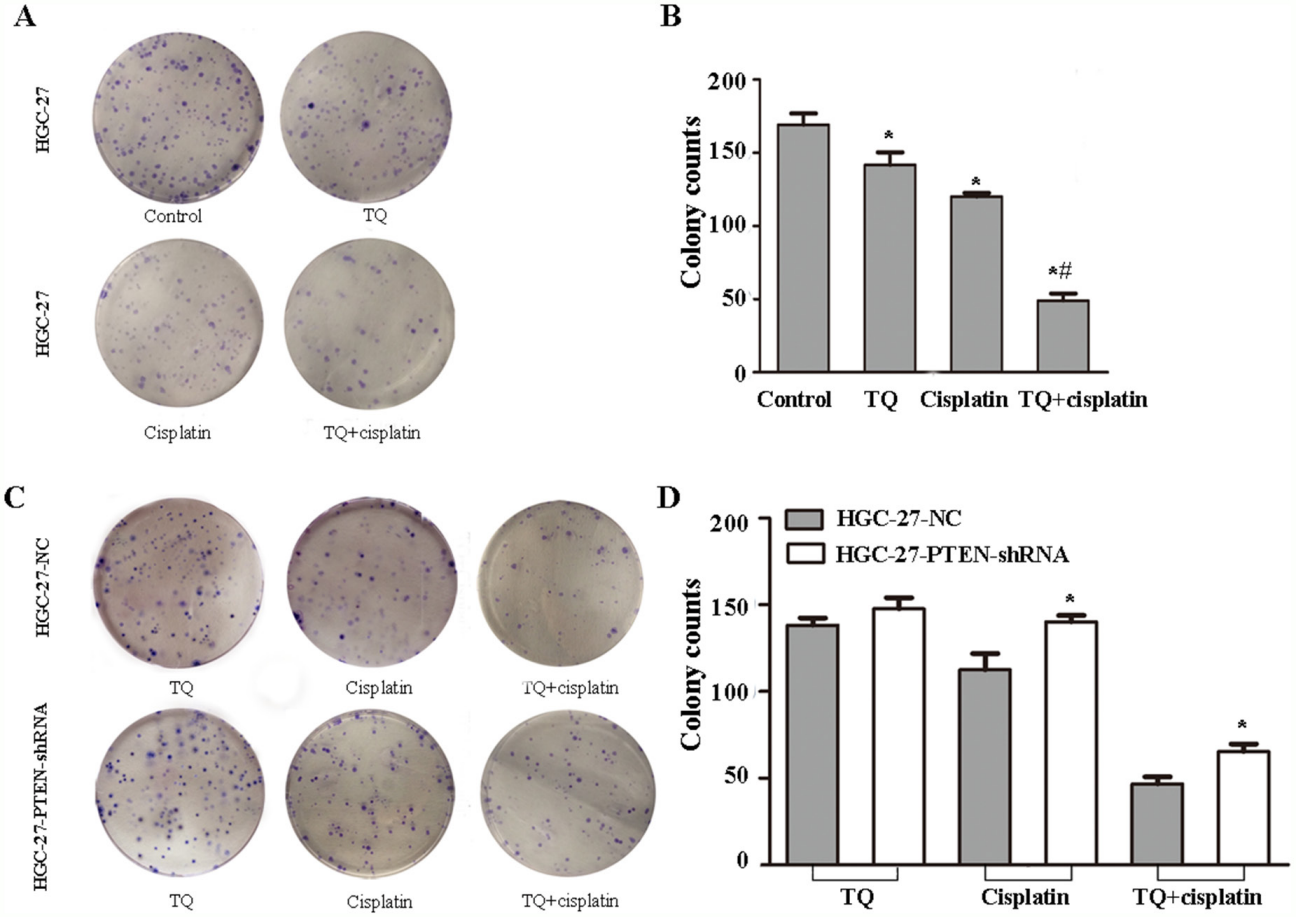
Effects of TQ and/or cisplatin on inhibiting the proliferation of GC cells and transfected GC cells by colony information assay **(A)** HGC-27 was treated with control, TQ (5μM), cisplatin (2μg/ml), the combination of 5μM TQ + 2μg/ml cisplatin. **(B)** Quantitative analysis of mean colony counts in each group in (A). ^*^P<0.05 versus untreated control cells, ^*#^*P*<0.05 versus single 2μg/ml cisplatin cells **(C)** Transfected HGC-27/PTEN-shRNA and HGC-27/NC were incubated with TQ (5μM), cisplatin (2μg/ml), the combination of 5μM TQ + 2μg/ml cisplatin, respectively. **(D)** Quantitative analysis of mean colony counts in each group in (C).^*^P<0.05 versus NC cells. All the above data are mean±SD from the average of three experiments.

**Figure 3:**
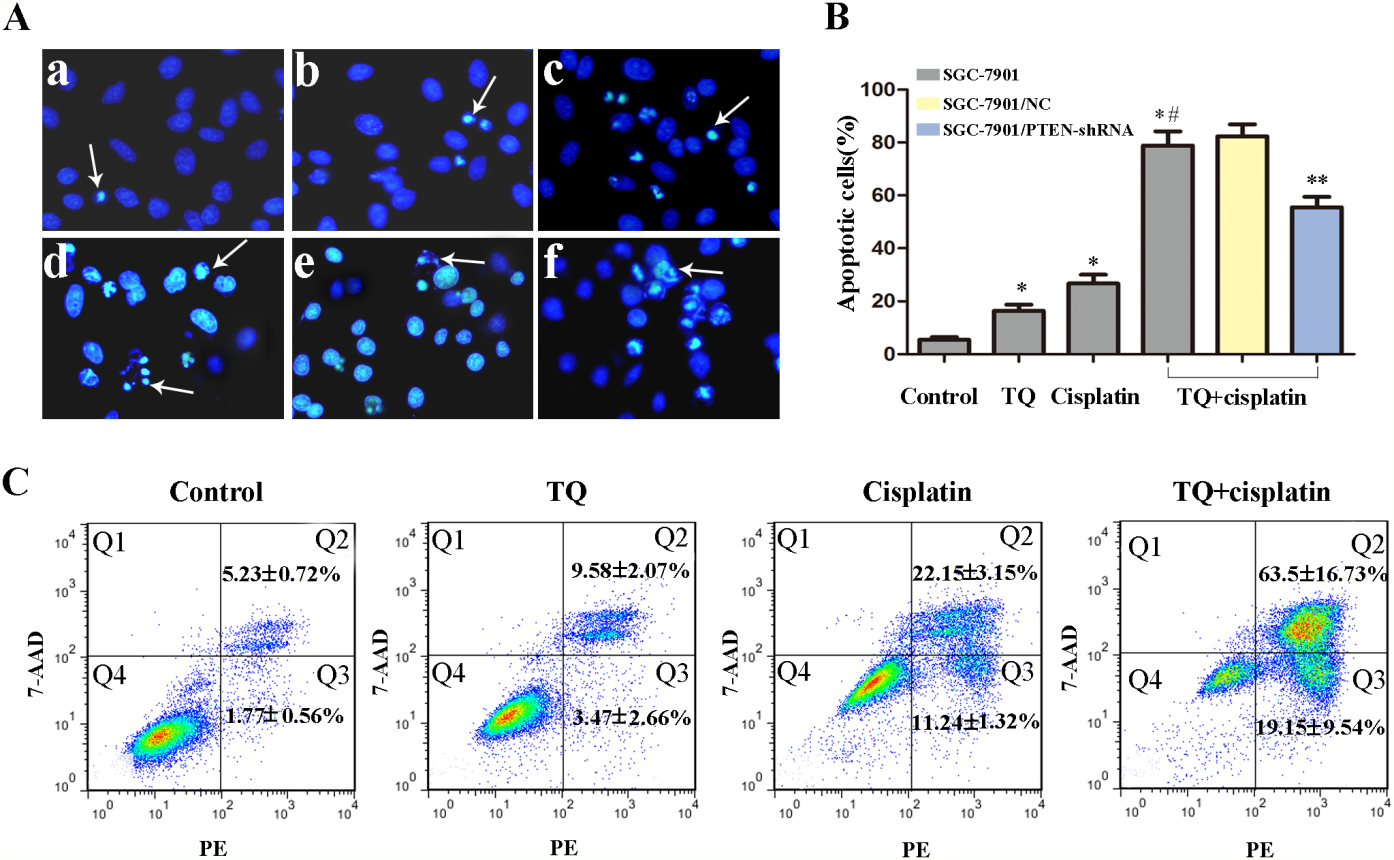
Effects of TQ and/or cisplatin on inducing apoptosis of GC cells by Hoechst 33258 and flow cytometry **(A)** Original magnification: 200×. SGC-7901 cells were incubated with TQ and/or cisplatin as following (a-d): (a) control, (b) 5μM TQ, (c) 2μg/ml cisplatin, (d) 5μM TQ +2μg/ml cisplatin. (e) 5μM TQ +2μg/ml cisplatin in SGC-7901/NC cells, (f) 5μM TQ +2μg/ml cisplatin in SGC-7901/PTEN-shRNA cells. The typical apoptosis cells were shown by arrows. **(B)** Quantitative analysis of apoptotic cells rate in each group in A. Bar graph for the apoptosis rate in 2μg/ml cisplatin, 5μM TQ, combination of 5μM TQ +2μg/ml cisplatin.^*^P< 0.05 versus untreated cells; ^*#^*P*< 0.05 versus single 2μg/ml cisplatin cells. ^**^P< 0.05 versus SGC-7901/NC cells. **(C)** Detection of apoptosis rate of SGC-7901 cells via Annexin V-PE/7-AAD double staining. All the above data are mean±SD from the average of three experiments.

**Figure 4:**
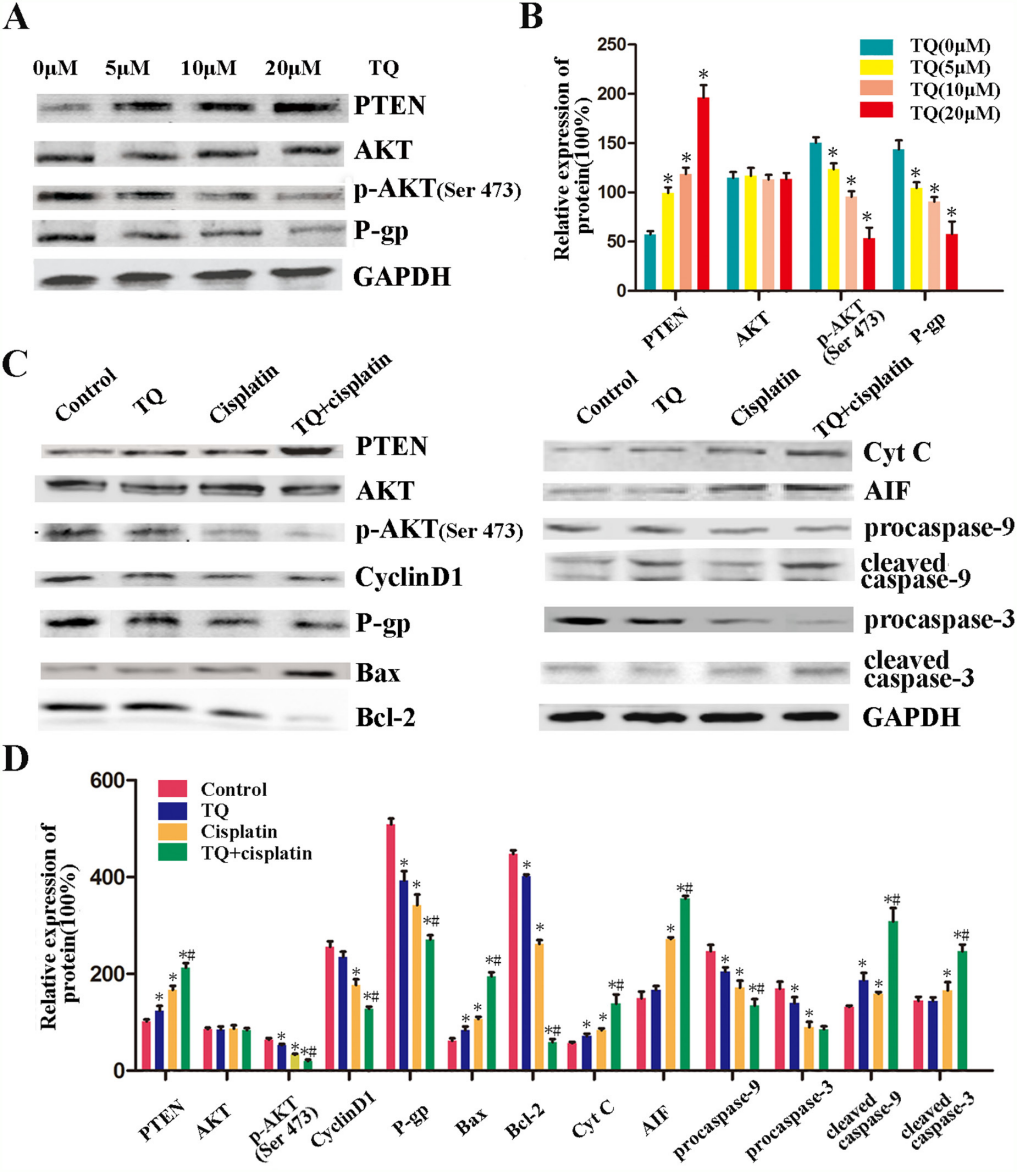
Effects of TQ and/or cisplatin on the changing levels of proteins on GC cells by western blotting **(A)** The expression levels of PTEN, AKT, p-AKT, P-gp proteins in SGC-7901 cells were detected. SGC-7901 cells were treated with TQ(0, 5, 10, 20μM) for 24h. **(B)** Quantitative analysis of proteins in A. ^*^P <0.05 versus control. **(C)** The expression levels of PTEN, AKT, p-AKT, CyclinD1, P-gp, Bax, Bcl-2, Cyt C, AIF, Apaf-1, procaspase-9, cleaved caspase-9, procaspase-3, cleaved caspase-3 in SGC-7901 cells were detected. SGC-7901 cells were incubated with 5μM TQ, 2μg/ml cisplatin, and 5μM TQ pretreated +2μg/ml cisplatin as described above. **(D)** Quantitative analysis of proteins in C.^*^P <0.05 versus untreated cells,^*#^ *P*< 0.05 versus cells treated with cisplatin alone. All the experiments are done three times. The above data are expressed as mean±SD.

**Figure 5:**
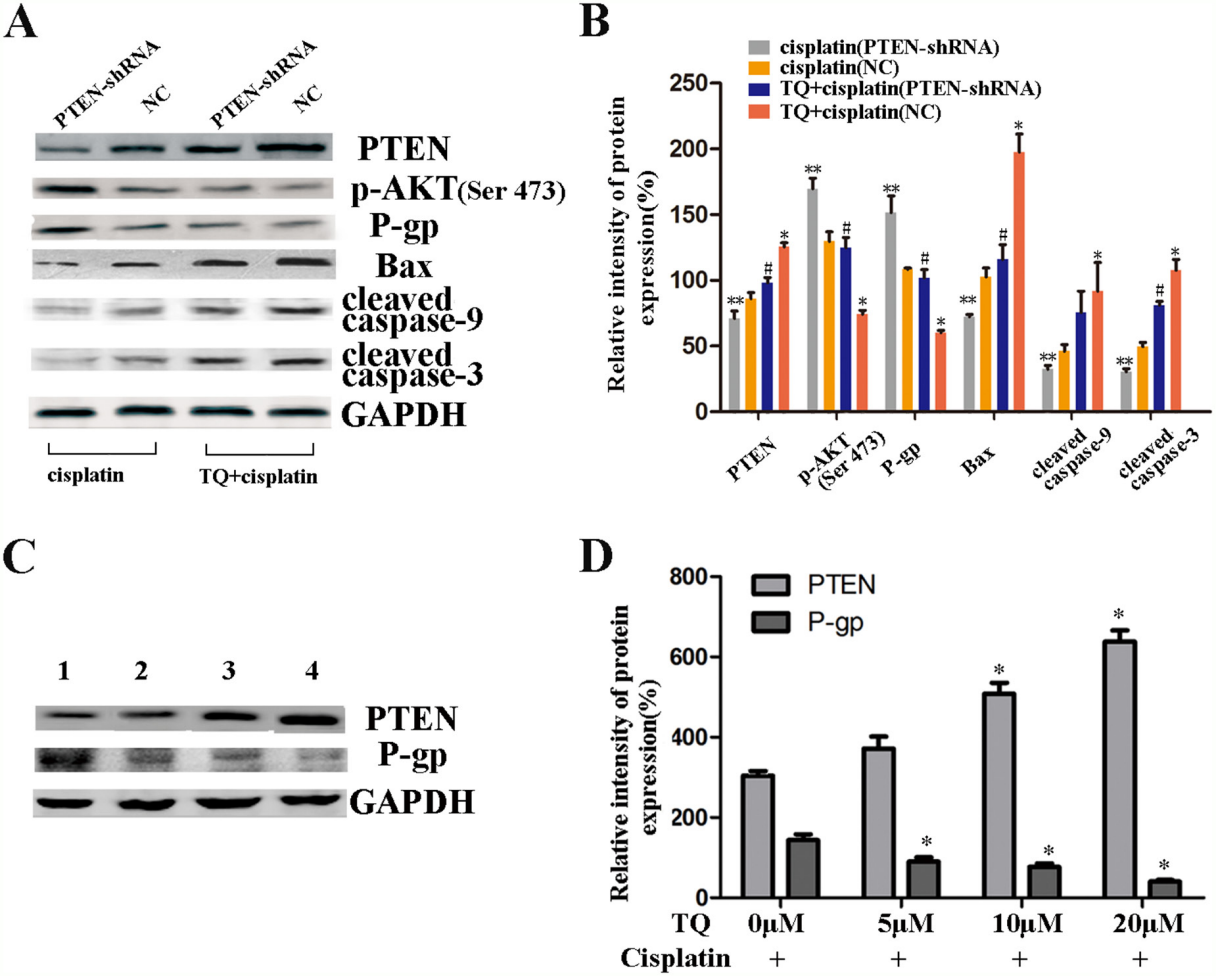
Western blotting further showing the contribution of PTEN gene to the apoptotic effects of cisplatin and a combination of TQ and cisplatin on GC cells **(A)** The expression levels of PTEN, p-AKT, P-gp, Bax, cleaved caspase-9, and cleaved caspase-3 in SGC-7901/NC, and SGC-7901/PTEN-shRNA cells which were incubated with 2μg/ml cisplatin alone, and a combination of 5μM TQ + 2μg/ml cisplatin, respectively. **(B)** Quantitative analysis of proteins in A.^*^P<0.05 versus NC cells intubated with 2μg/ml cisplatin alone, ^#^*P*<0.05 versus NC cells incubated with 5μM TQ + 2μg/ml cisplatin, **P<0.05 versus NC cells intubated with 2μg/ml cisplatin alone. **(C)** The expression levels of PTEN and P-gp proteins in SGC-7901 cells which were pretreated with TQ + cisplatin, respectively. Line 1, 0μM TQ+2μg/ml cisplatin; Line 2, 5μM TQ+2μg/ml cisplatin; Line 3, 10μM TQ+2μg/ml cisplatin; Line 4, 20μM TQ+2μg/ml cisplatin. **(D)** Quantitative analysis of proteins in C. ^*^P<0.05 versus 0μM TQ + 2μg/ml cisplatin. All the experiments are done three times. The above data are expressed as mean±SD.

**Figure 6:**
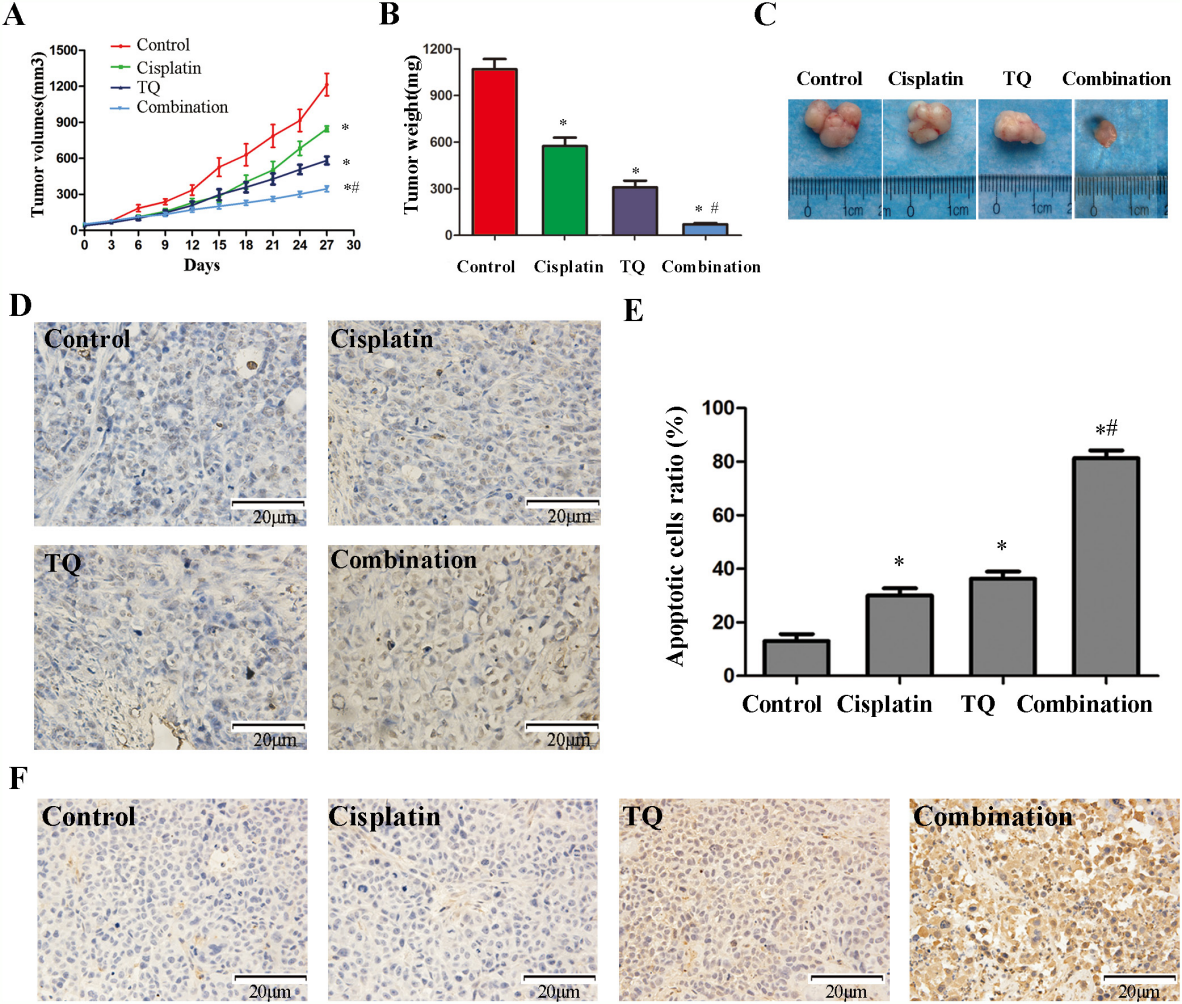
Anti-tumor effects of TQ and cisplatin in a gastric tumor xenograft mouse model **(A)** Each time point represents the mean tumor volume for each group. **(B)** Tumor weight was obtained at the end of the experiment. Error bars represent the standard error of the mean (SEM). **(C)** Xenograft tumors in each group were harvested and extracted completely after 30 days. **(D)** Detection of apoptotic cells in tumor tissue by TUNEL assay. **(E)** Quantitative analysis of apoptotic cells in D. *P<0.05 versus control group, *#*P*<0.05 versus cisplatin alone group. **(F)** The apoptotic cells per field in each group were counted on the basis of viewing eight random fields in each glide.

**Table 1:** Summary of IC50 values of cisplatin in GC cells^a^.

Cell type	IC50±SD(μg/ml)^b^	
	cisplatin	TQ+cisplatin
SGC-7901	1.78±0.17	0.96±0.02^*^
HGC-27	2.01±0.37	1.39±0.36^*^
MGC-803	1.72±0.02	1.09±0.09^*^

^a^ Cells were treated with cisplatin (0, 0.25, 0.5, 1, 2, 4 μg/ml) and a combination of 5μM TQ pretreatment + cisplatin (0, 0.25, 0.5, 1, 2, 4 μg/ml) for 24h, respectively.

^b^ All the above data are shown as the mean ± standard deviation (SD) of from the average of three experiments.

^*^ *P*<0.05 for values versus that obtained in corresponding GC cells treated with cisplatin alone.

GC cells were conducted stable transfection with PTEN-shRNA for down-regulation of PTEN, and NC (negative control) plasmid. The transfected efficiency was accessed by western blotting and shown in the Figure 1D. Transfected GC cells were treated TQ (5μM), cisplatin (0, 0.25, 0.5, 1, 2, 4μg/ml), and a combination of TQ(5μM) pretreatment + cisplatin(0, 0.25, 0.5, 1, 2, 4μg/ml), respectively. For TQ(5μM) group, although a slight increase in cell viability in GC cells transfected with PTEN-shRNA was found compared with NC group, there was no significant difference (*P*>0.05) (Figure 1E). However, TQ might augment cisplatin-induced growth inhibition, whereas down-regulation of PTEN might reverse this kind of combined effect of TQ and cisplatin on GC cells (Figure 1F). Furthermore, shown in the Table 2, our results revealed that TQ pretreatment following cisplatin, contributed to a decrease in the IC50 values of cisplatin, whereas down-regulation of PTEN might reverse this contribution, meanwhile, down-regulation of PTEN seemed to reduce the sensitivity of GC cells to cisplatin.

**Table 2:** Summary of IC50 values of cisplatin in transfected GC cells^a^.

Cell type	Transfection	IC5±SD(μg/ml)^b^	
		cisplatin	TQ+cisplatin
SGC-7901	NC	1.43±0.04	0.90±0.11^*^
	PTEN-ShRNA	1.81±0.22^&^	1.32±0.11^#^
HGC-27	NC	1.76±0.09	1.16±0.12^*^
	PTEN-ShRNA	2.21±0.31^&^	1.49±0.28
MGC-803	NC	1.77±0.07	0.99±0.09^*^
	PTEN-ShRNA	1.99±0.25	1.46±0.04^#^

^a^ Transfected GC cells were treated with cisplatin (0, 0.25, 0.5, 1, 2, 4 μg/ml) and a combination of 5μM TQ pretreatment + cisplatin (0, 0.25, 0.5, 1, 2, 4 μg/ml) for 24h, respectively.

^b^ All the above data are shown as the mean ± standard deviation (SD) of from the average of three experiments.

^*^ *P*<0.05 for values versus that obtained in corresponding NC cells treated with cisplatin alone.

^#^ *P*<0.05 for values versus that obtained in corresponding NC cells treated with TQ pretreatment following cisplatin.

^&^ *P*<0.05 for values versus that obtained in corresponding NC cells treated with cisplatin alone.

In the colony formation, the numbers of HGC-27 cells colony in control, cisplatin(2μg/ml), TQ(5μM), and TQ pretreatment following cisplatin were 169.3 ±10.6, 126.3 ±11.6, 120.0±3.6, 49±6.9, respectively (Figure 2A, 2B). Furthermore, transfected HGC-27 cells were treated with TQ(5μM), cisplatin(2μg/ml), and TQ(5μM)+cisplatin(2μg/ml), respectively. As shown in the Figure 2C, down-regulation of PTEN in HGC-27 cells caused an increase in the numbers of colony in cisplatin, and TQ+cisplatin group (^*^P<0.05, respectively). These results further approved that cells pretreated with TQ were more sensitive to cisplatin, and down-regulation of PTEN expression might weaken cisplatin-induced growth inhibition, and reverse the phenomenon of combined effect of TQ and cisplatin on GC cells.

### TQ augments cisplatin-induced apoptosis in GC cells, which was associated with PTEN gene

The morphological features of apoptotic cells were detected by Hoechst 33258 staining. Normal-blue fluorescence were found in normal cells, whereas condensed nuclei and karyorrhexis with bright-blue fluorescence were found in apoptosis cells. The random field per dish were carefully observed and further counted under a fluorescence microscope. As shown in the Figure 3, significant differences were found in the number of apoptotic cells, the percentage of apoptotic cells induced by TQ and/or cisplatin was significant higher than that of the untreated control group (^*^P< 0.05 versus untreated cells). Furthermore, the apoptotic cells of the combined treatment was higher than those of cisplatin alone (^*#^*P*< 0.05 versus single 2μg/ml cisplatin cells, Figure 3B). However, for the combination group, the apoptosis rate of GC cells transfected with PTEN-shRNA which knocked down PTEN expression, was significantly lower than that of NC group (^**^P< 0.05 versus NC cells, Figure 3B). The results indicated that TQ might augment cisplatin-induced apoptosis in gastric cancer cells, which might be associated with PTEN gene.

Apoptosis induced by TQ and cisplatin was further confirmed by Annexin PE/7-AAD staining. SGC-7901 cells treated with a combination (5μM TQ + 2ug/ml cisplatin) contained more early and late apoptotic cells (19.15% and 63.5%) than control (1.77% and 5.23%), TQ (3.47% and 9.58%), cisplatin (11.24% and 22.15%), which further revealed that TQ augments cisplatin-induced apoptosis in gastric cancer cells (Figure 3C).

### TQ sensitizes GC cells to cisplatin through the negative regulation of PI3K/AKT signaling pathway and activation of the mitochondrial pathway by up-regulating PTEN expression

To further explore the mechanisms of TQ sensitizing GC cells to cisplatin, western blotting was done to detect the related protein expression. PI3K/AKT signaling pathway is an important signaling pathway controlling cancer cell proliferation. We focus on the mechanisms of TQ-induced cytotoxicity effects on GC cells. SGC-7901 cells were treated with TQ(0, 5, 10, 20μM) for 24h. As shown in the Figure 4A, 4B, TQ led to an obvious increase in PTEN proteins, and a clear decrease in p-AKT and P-gp proteins in a concentration-dependent manner, which indicated that TQ might down-regulate the expression of P-gp through negative regulation PI3K/AKT signaling pathways by up-regulating PTEN gene.

We further explored the mechanisms of a combined effects of TQ and cisplatin on GC cells. SGC-7901 cells were incubated with 5μM TQ, 2μg/ml cisplatin, and 5μM TQ pretreatment +2μg/ml cisplatin as described above. As shown in Figure 4C, 4D, we found the expression of PTEN was obviously increased by the combination of TQ and cisplatin. Although no significant difference was found about the expression of total AKT, the expression of p-AKT was inhibited clearly by cisplatin, and TQ further potentiated this effect. Meanwhile, as the downstream important cell cycle protein of PI3K/AKT signaling pathway, CyclinD1 was also inhibited as p-AKT. Additionally, TQ and cisplatin led to an unbalance of anti-apoptotic/pro-apoptotic (Bax/Bcl-2) proteins, since they caused an increase in Bax protein levels, and a decrease in Bcl-2 protein levels. An obvious increase in apoptotic AIF, and Cyt C, cleaved caspase 9, and cleaved caspase 3 proteins, and a decrease in procaspase 9 and procaspase 3 were also found in combination group compared with that in cisplatin alone. These above results indicated that TQ sensitizes GC cells to cisplatin, playing anti-tumor effects on GC cells through the negative regulation of PI3K/AKT signaling pathway and activation of the mitochondrial pathway by up-regulating PTEN expression.

### Down-regulation of PTEN gene contributes to weakening the apoptosis induced by cisplatin alone and a combination of TQ and cisplatin, respectively

We further assessed the contribution of PTEN gene to the apoptotic effects of cisplatin and a combination of TQ and cisplatin on GC cells. The cells were transfected with PTEN-shRNA and NC, and transfection efficiency of PTEN was assessed by western blotting (Figure 1D). SGC-7901/NC, and SGC-7901/PTEN-shRNA were incubated with 2μg/ml cisplatin alone, and a combination of 5μM TQ + 2μg/ml cisplatin, respectively. As shown in Figure 5A, TQ and cisplatin led to a clear increase in PTEN protein, an obvious decrease in p-AKT, P-gp proteins, and a clearly increase in apoptotic proteins Bax, cleaved-caspase-9, and cleaved-caspase-3 protein than cisplatin alone in NC cells (^*^P<0.05 versus cisplatin alone, Figure 5B). However, as also shown in Figure 5A, down-regulation of PTEN caused an apparent increase in p-AKT and P-gp, and a clear decrease in apoptotic proteins Bax, cleaved-caspase-9, and cleaved-caspase-3 compared with NC cells treated with a combination of 5μM TQ + 2μg/ml cisplatin or cisplatin alone, respectively (^#^*P*<0.05 versus NC cells incubated with TQ + cisplatin, **P<0.05 versus NC cells incubated with cisplatin alone, Figure 5B). All the above results seem to demonstrate that TQ augments cisplatin-induced apoptosis on SGC-7901/NC cells via targeting PTEN gene negatively regulating PI3K/AKT signaling pathways, activating mitochondrial apoptosis pathway and importantly, down-regulation of PTEN gene contributes to weakening the apoptosis induced by cisplatin alone and a combination of TQ and cisplatin, respectively.

### TQ enhanced cisplatin-induced inhibitory effects on GC cells through down-regulation of P-gp by up-regulating PTEN expression

Our above results revealed that TQ might up-regulate PTEN gene on GC cells, which seems to cause a clearly decrease in the expression of P-gp. Firstly, TQ led to an obviously increase in PTEN proteins, and a clearly decrease in p-AKT and P-gp proteins in SGC-7901 cells, in a concentration-dependent manner (Figure 4A, 4B). Furthermore, TQ and cisplatin led to a clear increase in PTEN proteins, in contrary, a significant decrease in p-AKT and P-gp protein levels in SGC-7901 cells. Additionally, to further investigate whether the combined inhibitory effects of TQ and cisplatin on GC cells was associated with down-regulation of P-gp by PTEN, SGC-7901/NC, and SGC-7901/PTEN-shRNA were treated with a combination of TQ and cisplatin as described above. As shown in the Figure 5A, followed the down-regulation of PTEN, an obvious increase in p-AKT and P-gp protein levels were found. Interestingly, as shown in the Figure 5C, SGC-7901 cells were treated with a combination of TQ (0, 5, 10, 20μM) + cisplatin (2ug/ml) for 24h respectively, the expression of PTEN was significantly increased, meanwhile, the expression of P-gp was also been measured. An obvious decrease in the P-gp proteins was observed and quantified. Therefore, we proposed that the combined inhibitory effects of TQ and cisplatin on GC cells was associated with down-regulation of P-gp by up-regulating PTEN.

### Anti-tumor effects of TQ and cisplatin on gastric cancer cells *in vivo*

On the basis of the data *in vitro* above, we further investigated the effects of TQ and/or cisplatin on xenograft tumor growth *in vivo*. As shown in the Figure 6A, tumors grew progressively and reached approximately 1214.92±207.99 mm^3^ in control group. However, tumor growth was suppressed by TQ and cisplatin *in vivo*, respectively (582.49 ±75.43mm^3^ and 845.01±53.09mm^3^) (^*^P<0.05 versus control). Furthermore, the combination of TQ and cisplatin significantly inhibited tumor growth *in vivo* (345.99±54.83 mm^3^) compared with cisplatin (^*#^*P*<0.05 versus cisplatin alone). Meanwhile, at the end of experiment, the tumors were all harvested. The weight of the tumors in control group was heavier than that of the tumors in cisplatin (5mg/kg) group, TQ (5mg/kg) and combination (TQ 5mg/kg+cisplatin5mg/kg) group, furthermore, tumors growth were significantly inhibited in combination group than cisplatin (5mg/kg) group(^*#^*P*<0.05) (Figure 6B).

Tumor tissues isolated from the xenograft mice of four groups were processed for HE staining (not shown), TUNEL assay, and IHC for the detection of PTEN protein. As shown in the Figure 6D, the combination of TQ and cisplatin resulted in an apparent increase in cell apoptosis in the tumor mass (^*#^*P*<0.05, Figure 6E), which indicated that TQ augments cisplatin-induced apoptosis in tumors *in vivo*. Additionally, the expression of PTEN protein by IHC showed that TQ augments cisplatin-induced anti-tumor effects by up-regulating PTEN expression *in vivo* (Figure 6F).

## DISCUSSION

Although cisplatin has yielded clinical benefits for advanced gastric cancer for decades, the overall outcome remains poor mainly due to drug resistance, posing a major clinical challenge without doubt [17]. Complex mechanisms contribute to the development of drug resistance. MDR is a major cause of failure in cancer chemotherapy. The overexpression of a series of trans-membrane proteins, especially P-gp, plays a key role in producing MDR in human cancer via extruding intracellular anti-cancer drugs and thus decreasing drugs accumulation [18, 19]. It is a feasible strategy to develop new cancer chemopreventive or chemotherapeutic agent to reverse MDR for chemotherapy.

Previous studies has identified that a kind of Chinese herb enhanced the apoptosis induced by conventional chemotherapeutic drugs on human cancers both *in vivo* and *in vitro* [20, 21]. However, the specific mechanism is still unclear. Our research team has demonstrated that increasing TQ concentration inhibits proliferation of gastric cancer cells *in vivo* and *in vitro* [16]. It has been reported that TQ acts as a booster for the anti-cancer effects of doxorubicin, gemcitabine and oxaliplatin in cancer cell lines [22, 23]. Interestingly, a recent study reports that the combination of cisplatin and TQ is highly effective in non-small cell lung cancer (NSCLC); this combination contributes to overcoming the cisplatin resistance [24]. However, both the sensitization of TQ to cisplatin and the specific mechanism in GC have not been clarified.

Our study found TQ and cisplatin can inhibit the proliferation of GC cells in a concentration-dependent manner, respectively (Figure 1A, 1B). A pre-treated concentration TQ 5μM, was identified since cell viability at this concentration reached approximately 90%, with no obvious cytotoxicity in all GC cell lines tested. TQ(5μM), sensitizes GC cells to cisplatin-induced growth inhibition (Figure 1B, 1C, 2A and Table 1). Meanwhile, TQ(5μM) significantly augments cisplatin-induced apoptosis on GC cells (Figure 3A-3C). Additionally, in human xenograft tumor models in nude mice, TQ and cisplatin led to inhibiting tumor growth significantly compared with TQ or cisplatin alone (Figure 6A-6C), increasing apoptosis induced by cisplatin was another feature of transplanted tumor tissue response to combined TQ and cisplatin treatment (Figure 6D, 6E). Meanwhile, the over-expression of PTEN, induced by the combination of TQ and cisplatin was further conformed through IHC *in vivo* (Figure 6F). These results suggest that TQ seems to be a promising anti-cancer agent as a combination of conventional chemotherapeutic drugs cisplatin for GC treatment.

On the basis of mentioned results above, we deeper explored the underlying mechanisms which TQ augments cisplatin-induced anti-tumor effects on human gastric cancer cells. In our study, we found that TQ inhibits the proliferation of gastric cancer cells via negatively regulating PI3K/AKT signaling pathways by up-regulating PTEN (Figure 4A, 4B).

Similar to our findings, a recent research has clarified that TQ induces apoptosis in doxorubicin-resistant breast cancer cells through up-regulation of PTEN at transcription level [15]. It has been reported that PTEN negatively regulates the PI3K/AKT signaling pathway, leading to substantially decreasing p-AKT, controlling cancer cell proliferation [25]. Interestingly, another research reported that Tangeretin sensitizes cisplatin-resistant human ovarian cancer cells through down-regulation of PI3K/Akt signaling pathways [26]. The combination of TQ and gemcitabine contributes to increasing apoptosis and inhibiting tumor growth in pancreatic cancer via inactivation of Akt/mTOR/S6 signaling pathways by up-regulation of PTEN [27]. Therefore, it is quite reasonable to assume that PTEN gene might play a key role in TQ sensitizing gastric cancer cells to cisplatin.

We detected the levels of a series of important proteins in GC cells after being incubated with 5μM TQ, 2μg/ml cisplatin, and 5μM TQ pretreated +2μg/ml cisplatin as described above. Importantly, an obvious increase in the levels of PTEN, and a decrease in the levels of p-AKT, CyclinD1 were found in TQ + cisplatin, compared with TQ or cisplatin alone. Previous studies reported that Bcl-2 family proteins were one of the components of mitochondrial permeability transition pore (mPTP) [28]. The structure and permeability of mPTP will be changed when Bcl-2/Bax protein ratio is reduced [29]. To clarify whether the up-regulation of PTEN by TQ and cisplatin led to the apoptosis of gastric cancer cells via mitochondrial pathway, we detected the levels of Bcl-2, Bax, procaspase-9, procaspase-3, Cyt C, AIF, cleaved caspase-9, cleaved caspase-3 using western boltting. It was found that TQ and cisplatin caused an increase in the levels of Bax, Cyt C, AIF, cleaved caspase-9, and cleaved caspase-3 and a decrease in Bcl-2, procaspase-9, procaspase-3 levels (Figure 4C, 4D). These results indicated that TQ sensitizes GC cells to cisplatin, playing anti-tumor effects on GC cells through the negative regulation of PI3K/AKT signaling pathway and activation of the mitochondrial pathway by up-regulating PTEN expression.

To clearly clarify whether PTEN gene plays a key role in TQ sensitizing gastric cancer cells to cisplatin, GC cells were conducted stable transfection with PTEN-shRNA for down-regulation of PTEN, and NC plasmid (Figure 1D). Transfected GC cells were treated TQ (5μM), cisplatin (0, 0.25, 0.5, 1, 2, 4μg/ml), and a combination of TQ(5μM)+ cisplatin(0, 0.25, 0.5, 1, 2, 4μg/ml), respectively. We found that TQ contributed to augmenting cisplatin-induced growth inhibition, whereas down-regulation of PTEN might reverse this combined effects of TQ and cisplatin on GC cells, and reduce GC cells to cisplatin-sensitivity (Figure 1F). Furthermore, as shown in the Figure 2C, 2D, the colony formation approved our above findings again. According to Hoechst 33258 staining, TQ pretreatment following cisplatin, synergistically increased apoptosis in gastric cancer cells, which could be weaken by down-regulation of PTEN (Figure 3A, 3B). Transfected SGC-7901 cells were treated with 2μg/ml cisplatin and 5μM TQ + 2μg/ml cisplatin, respectively. An apparent decline in apoptotic proteins Bax, cleaved-caspase-9, cleaved-caspase-3 was further found in SGC-7901/PTEN-shRNA cells compared with SGC-7901-NC (Figure 5A, 5B). Therefore, we demonstrated that PTEN gene plays a crucial role in TQ sensitizing gastric cancer cells to cisplatin.

Interestingly, our results also showed that, the combined inhibitory effects of TQ and cisplatin on GC cells was associated with down-regulation of P-gp by PTEN. TQ led to an obvious increase in PTEN proteins, and a clear decrease in p-AKT and P-gp proteins in SGC-7901 cells in a concentration-dependent manner (Figure 4A, 4B). TQ pretreatment following cisplatin also caused a clear increase in PTEN proteins, and, a significant decrease in P-gp protein levels (Figure 4B, 4C). However, an obvious increase in P-gp protein levels was found in GC cells transfected with PTEN-shRNA compared with NC, which were all treated with cisplatin, or TQ + cisplatin, respectively (Figure 5A, 5B). In addition, it’s interesting we observed that TQ achieved an increase in PTEN, and an obvious decrease in P-gp in a dose-dependent manner (Figure 5C, 5D). It has been reported that positive P-gp expression might act as an indicator of enhanced resistance to cisplatin *in vitro*, and efflux intracellular anti-cancer drugs thus decreasing anti-cancer drugs accumulation [19, 30]. Another study reported that, besides reduction of intracellular drug accumulation, constitutive activation of PI3K/Akt signals and dysfunction of the tumor suppressor gene p53 might contribute to cisplatin-resistance of cancer cells, which were regulated by PTEN gene [23, 31, 32]. According to above evidences, it is plausible to surmise that TQ sensitizes GC cells to cisplatin, the mechanism of which might be the increase in intracellular accumulation of cisplatin through down-regulation of P-gp controlled by TQ targeting PTEN. However, further studies are necessary to demonstrate that how the expression of P-gp is down-regulated by PTEN gene. Is it at the mRNA level, induced by protein degradation systems, via an unknown and important signaling pathway? We will further explore specific mechanism which are involved in the regulation of P-gp protein caused by controlled by TQ up-regulating PTEN.

In summary, our results provide strong molecular evidence in support of our hypothesis that TQ augments cisplatin-induced anti-tumor effects on gastric cancer cells via inhibiting PI3K/AKT signal pathway, activating of mitochondrial pathway, and down-regulating P-gp proteins by up-regulating PTEN gene both *in vitro* and *vivo*.

## MATERIALS AND METHODS

### Cell culture and regents

Human gastric cancer cells lines (SGC-7901, HGC-27, MGC-803) were donated by China Center for Type Culture Collection (CCTCC), and cultured in DMEM (HyClone) supplemented with 10% fetal bovine serum (FBS)(Gibco), 1% antibiotic solution (penicillin 100 U/ml and streptomycin 100 g/ml) (Beyotime, China) at 37°C, and 5% CO_2_ in a humidified incubator. TQ and cisplatin were obtained from Sigma-Aldrich. TQ was dissolved in absolute ethanol to prepare a 10 μM stock solution stored at -20°C. Cisplatin was dissolved in normal saline and stored at -20°C at a concentration of 4mg/ml. Lipofectamine® 2000 Transfection Reagent were purchased from Thermo Fisher Scientific, and stored at 4°C. Plasmid PTEN-shRNA (PTEN-RNAi-38319) and PTEN-NC (negative control, CON077) stored in glycerol bacteria(-20°C) was purchased from Shanghai Genechem biotechnology company (China).

### Transfection

Plasmid PTEN-shRNA and PTEN-NC with Green fluorescent protein (GFP) and anti-puromycin gene site, were extracted and further purified from glycerol bacteria according to the manufacturer’s instructions. Cells were seeded into a six-well plate, cultured in DMEM/F-12 with 10% FBS until a density of 70%-80% fusion, and then experienced with a hungry process in DMEM/F-12 without FBS for 2h. According to the manufacturer’s instructions, cells were transfected with 4μg of PTEN-shRNA or PTEN-NC using Lipofectamine 2000. After 24h, transfected cells were supplemented with DMEM/F-12 with 10% FBS and 0.5 u/ml puromycin for stable transfection. Ultimately, HGC-27/SGC-7901-PTEN, and HGC-27/SGC-7901-NC were successfully obtained. The down-regelation of PTEN expression was quantified by western blot analysis. Additionally, the transfection efficiency was indirectly observed by GFP using an inverted fluorescence microscope.

### Cell proliferation assay

Cell viability was evaluated quantitatively using Cell Counting Kit-8(CCK-8) purchased from Beyotime. All GC cells were seeded into 96-well plates (5×10^3^/plate). Firstly, after exposure to different concentrations of TQ (5, 10, 20, 40, 80μM), or cisplatin (0.25, 0.5, 1, 2, 4μg/ml) in DMEM for 24 hours, 10 μl CCK-8 solution was added to each well. Secondly, GC cells were plated and incubated with medium containing TQ (5μM), cisplatin (2μg/ml) and the combination of TQ and cisplatin, besides, HGC-27/SGC-7901-PTEN, and NC group cells were also exposed to the combination of TQ and cisplatin for 24 h, and the effect on cell viability was examined by CCK-8 as described above.

Colony information assay was conducted to evaluate cell proliferation. Cells or stably transfected cells were seeded (1×10^3^ cells/well) in 6-well plates. GC cells and transfected HGC-27 cells were respectively exposed to TQ (5μM) and/or cisplatin (2μg/ml) for 24h. The supernatant was replaced every 4 days, and the above processes were repeated. 14 days later, the cells were fixed by 4% paraformaldehyde and stained by Giemsa and colony numbers were counted.

### Hoechst 33258 assay for apoptosis

Hoechst 33258 Staining Kit (Beyotime) was used to detect the apoptotic morphological features. Cells in exponential growth was seeded into a six-well plate (1×10^5^ cells/well). GC cells were cultured into a six-well plate for 24h, and treated with TQ and cisplatin as mentioned above, and further stained with Hoechst 33258. Additionally, transfected GC cells were also exposed to the combination of TQ and cisplatin for 24h and stained as described above. Apoptotic morphological features was observed and captured using a fluorescent microscope (BX51, Olympus).

### Annexin V-PE/7-AAD double staining assay for apoptosis

Annexin V-PE/7-AAD kit (MultiSciences) was used to quantify the percentage of apoptotic cells by flow cytometry (FACSCalibur, Becton Dickinson). Cells were seeded into a six-well plate and incubated for 24h with either TQ, cisplatin or TQ and cisplatin. Adherent cells were collected and co-stained with 5-μl Annexin V-PE and 10-μl 7-AAD prior to flow cytometric analysis.

### Western blotting

Protein expression levels were assessed by Western blot analysis. GC cells were grown into a six-well plate for 24h, and treated with TQ, and cisplatin as mentioned above. Total proteins were extracted from the human GC cells or subcutaneous tumor tissues in nude mice. BCA Protein Assay Kit (Beyotime) was used to detect protein concentrations. The cellular proteins were all subjected to SDS-PAGE, transferred to polyvinylidene difluoride (PVDF) membranes (Millipore), and blocked with 5% non-fat dry milk in TBS. Then, the membranes were incubated with several primary rabbit antibodies at 4°C overnight. After washing with TBST three times (10min/time), membranes were further immunoblotted with secondary antibody for 1 h at room temperature. Washing the membranes three times again. Finally, the membranes were scanned by a two-color Odyssey infrared imaging system (LI-COR Biosciences). The specific protein expression levels were normalized to GAPDH on the same PVDF membranes.

### Xenograft tumor experiment in nude mice

All procedures performed in studies involving animal work were approved by Ethics Committee of Renmin Hospital of Wuhan University, which were in accordance with the ethical standards of the institutional and/or national research committee and with the 1964 Helsinki declaration and its later amendments or comparable ethical standards. Male BALB/c nude mice, 5-week-old, were purchased from Beijing Vital River Laboratory Animal Technology (China). Harvested GC cells were washed in serum-free DMEM, suspended in 100μ PBS, and implanted subcutaneously into the dorsal area of the nude mice. When the tumors approximately reached 100-150mm^3^ in size, the nude mice were randomly divided into four groups (six in each group) and received intraperitoneal injection of normal saline, TQ(5mg/kg), cisplatin(5mg/kg), and combination group (TQ 5mg/kg + cisplatin 5mg/kg), respectively every 2 days. During the treatment, tumor size was measured in two dimensions using vernier caliper by two researchers (2-3 times/week), and the tumor volume (TV) was calculated using the formula: TV (mm^3^)=0.5×d^2^×D, where d and Dare the shortest and longest diameters, respectively. Also, all the mice were weighed (2-3 times/week). Following the 30 days treatment, tumors were harvested, weighed, and then were analyzed by HE staining and TUNEL assay. Liver and renal function were measured through detection of the levels of alanine aminotransferase (ALT), aspartate aminotransferase (AST), blood urea nitrogen (BUN) and serum creatinine (Cr).

### HE staining, TUNEL assay and IHC

The paraffin tissues were prepared from tumor tissues in mice and cut into 4-μM-thick sections, and then stained with hematoxylin and eosin (HE). TUNEL assay was conducted to detect apoptotic cells using an *in situ* apoptosis detection kit (Roche Diagnostics). The specimens were observed using a light fluorescence microscope (OLYMPUS). The positive cells were identified, counted and analyzed. Additionally, Immunochemical (IHC) reactions was developed using an UltraSensitiveTM SP kit and DAB kit (China Fuzhou Maixin Biotech) according to the manufacturer’s instructions. The expression of PTEN in subcutaneous tumor tissues in mice was detected by IHC.

### Statistical analysis

SPSS software version 20.0 was used to for all the analyses. Data was expressed as mean±SD. The difference among groups was determined by ANOVA. A P-value of less than 0.05 was considered statistically significant.

## Abbreviations

GC: gastric cancer
TQ: Thymoquinone
P-gp: P-glycoprotein
NC: negative control
